# Psychophysiological responses of junior orienteers under competitive pressure

**DOI:** 10.1371/journal.pone.0196273

**Published:** 2018-04-26

**Authors:** Claudio Robazza, Pascal Izzicupo, Maria Angela D’Amico, Barbara Ghinassi, Maria Chiara Crippa, Vincenzo Di Cecco, Montse C. Ruiz, Laura Bortoli, Angela Di Baldassarre

**Affiliations:** 1 Department of Medicine and Aging Sciences, “G. d’Annunzio” University of Chieti-Pescara, Chieti, Italy; 2 SPAEE, Service of Educational and Learning Psychology, “Sacro Cuore” Catholic University of Milan, Milan, Italy; 3 FISO, Italian Federation of Orienteering Sports, Trento, Italy; 4 Faculty of Sport and Health Sciences, University of Jyväskylä, Jyväskylä, Finland; Universita degli Studi di Verona, ITALY

## Abstract

The purpose of the study was to examine psychobiosocial states, cognitive functions, endocrine responses (i.e., salivary cortisol and chromogranin A), and performance under competitive pressure in orienteering athletes. The study was grounded in the individual zones of optimal functioning (IZOF) and biopsychosocial models. Fourteen junior orienteering athletes (7 girls and 7 boys), ranging in age from 15 to 20 years (*M* = 16.93, *SD* = 1.77) took part in a two-day competitive event. To enhance competitive pressure, emphasis was placed on the importance of the competition and race outcome. Psychophysiological and performance data were collected at several points before, during, and after the races. Results showed that an increase in cortisol levels was associated with competitive pressure and reflected in higher perceived exertion (day 1, *r* = .32; day 2, *r* = .46), higher intensity of dysfunctional states (day 1, *r* = .59; day 2, *r* = .55), lower intensity of functional states (day 1, *r* = -.36; day 2, *r* = -.33), and decay in memory (day 1, *r* = -.27; day 2, *r* = -.35), visual attention (day 1, *r* = -.56; day 2, *r* = -.35), and attention/mental flexibility (day 1, *r* = .16; day 2, *r* = .26) tasks. The second day we observed better performance times, lower intensity of dysfunctional states, lower cortisol levels, improved visual attention and attention/mental flexibility (*p* < .050). Across the two competition days, chromogranin A levels were higher (*p* < .050) on the most difficult loops of the race in terms of both physical and psychological demands. Findings suggest emotional, cognitive, psychophysiological, and performance variables to be related and to jointly change across different levels of cognitive and physical load. Overall results are discussed in light of the IZOF and biopsychosocial models. The procedure adopted in the study also supports the feasibility of including additional cognitive load for possible practical applications.

## Introduction

The interplay between emotion and cognition under pressure has recently attracted research interest [1]. A leading perspective to the study of emotions in sport is the individual zones of optimal functioning (IZOF) model [2]. The model provides a holistic perspective in the description of subjective emotion and non-emotion performance-related states (i.e., psychobiosocial states). The main dimensions that define the structure of a performance-related psychobiosocial state are form, content, and intensity. The form dimension refers to the multimodal display of performance-experiences in a wide range of specific and interrelated psychobiosocial states. The content dimension involves the functionality‒hedonic tone interplay that leads to functional or dysfunctional states for performance perceived as pleasant or unpleasant. The intensity dimension relates to the states amount or quantity. According to the tenets of the IZOF model [2], past, ongoing, and anticipated person-environment interactions are reflected in a variety of psychobiosocial states. These functional/dysfunctional, pleasant/unpleasant states are manifested in psychological (i.e., affective, cognitive, motivational, volitional), biological (i.e., bodily-somatic, motor-behavioral), and social (i.e., operational, communicative) modalities [3, 4].

The relationship between psychobiosocial states and performance is assumed to be bi-directional, implying that psychobiosocial states can influence performance and, conversely, on-going performance can influence psychobiosocial states. Prior to and during performance, one’s appraisals of anticipated and current gains or losses tend to elicit challenge states (e.g., feeling confident) or threat states (e.g., feeling worried), respectively. Performance level is predicted based on the interaction of both functional (challenge) and dysfunctional (threat) states. High probability of successful performance is expected to occur when the athlete experiences high functional and low dysfunctional psychobiosocial states [2]. This multimodal view concurs with the biopsychosocial model of challenge and threat [5], which integrates biological (i.e., autonomic and endocrine influences on the cardiovascular system), psychological (i.e., affective and cognitive influences on evaluative processes), and social (i.e., person and environmental interplay) modalities to explain motivational processes of individual performance.

Both the IZOF and biopsychosocial models build upon Lazarus’ [6] appraisal theory of emotion. The theory draws on the notion that threatening situations involve the appraisal of potential for harm or loss, whereas challenging situations entail the appraisal of opportunities for growth, mastery, or gain. Emotional responses are also triggered by individual evaluation of available coping resources and response options. In motivated performance contexts, the interaction between appraisal of situational demands and coping resources elicits challenge and threat responses, which encompass a set of interrelated affective, cognitive, motivational, physiological, expressive or behavioral, and social components [2, 5, 7]. Challenge is experienced when the appraisal of personal coping resources meets or exceeds situational demands, whereas threat arises when perceived demands exceed resources. Extant research findings support the hypothesis that challenge states lead to superior athletic performance compared to threat states [8–11].

Distinct patterns of neuroendocrine and cardiovascular activity are postulated to reflect challenge or threat states in athletes [12]. According to this view, a challenge state is accompanied by increased epinephrine level and cardiac activity, reduced total peripheral vascular resistance, and either pleasant or unpleasant emotions experienced as helpful for performance. On the other hand, a threat state is associated with increased cortisol level, smaller increases in cardiac activity, stable or enhanced peripheral vascular resistance, and unpleasant emotions perceived as harmful [12–14]. Although research on the biopsychosocial mechanisms associated with performance in sport is still scant, a challenge state is suggested to determine positive consequences on performance deriving from improved decision making and cognitive functioning, enhanced task engagement, and less effort spent to self-regulation [12]. On the other hand, a challenge state is proposed to influence performance negatively due to decreased cognitive functioning and task involvement, and greater resources devoted to self-regulation.

Several interactive factors have been proposed to influence whether individuals feel that they have or not the resources to cope with a stressful situation and, therefore, to determine a challenge or threat state with the subsequent physiological and psychological responses [5, 14]. These antecedents include, among others, familiarity, required effort, skills, knowledge, and abilities. For example, high familiarity with a task, low required effort, and high skill levels are likely leading to one’s evaluation of a situation as a challenge instead of a threat. In contrast, low familiarity, high effort, and poor skills are expected to evoke evaluations of the situation in terms of a threat rather than a challenge. It should be noted that challenge and threat are not dichotomous states, but represent anchors along a bipolar continuum. Thus, researchers have often studied relative differences in challenge and threat rather than absolute differences [13, 14].

Grounded in the IZOF and biopsychosocial models, the purpose of this study was to examine psychobiosocial states, cognitive (executive) functions, endocrine responses (i.e., salivary cortisol and chromogranin A), and performance under pressure in orienteering sport, which involves highly physical, cognitive, and emotional demands. Orienteers need good aerobic fitness to engage in a foot race in a wild environment. Navigating on an unfamiliar terrain between a number of control locations in an established order with the help of a map and compass in the quickest time is also a cognitive challenge. The orienteers, indeed, are provided with the orienteering map just seconds before the beginning of the race. This implies that they plan a route from the map during the race. Successful performance requires considerable visual attention to critical cues from the map, the environment, and the travel [15]. Attending simultaneously to the three sources of information and making effective decisions under time constraints and competitive stress entails complex and dynamic processes of perception, encoding, retrieval, decision making, and emotion regulation. Thus, executive functions, such as focused attention, working memory, and cognitive flexibility, are essential in orienteering. These top-down control processes underlie higher order cognitive functions involved in goal-directed behaviors, such as problem-solving, decision making, and planning [16].

Working memory, in particular, refers to the limited capacity and multicomponent cognitive ability to retain in mind and manipulate complex information (i.e., verbal and visuospatial), no longer perceptually present, over short periods of time [16, 17]. It is critical for making sense of experiences that unfold over time (i.e., remembering what happened earlier and relating it to what comes later), and for different mental processes including attentional allocation (i.e., selectively attending to environmental stimuli and tuning out irrelevant stimuli) and switching between mental sets (i.e., cognitive flexibility). Working memory is reflective of one’s ability to focus on task goals, suppress interferences, and avoid distractions. Research evidence has shown correlations between working memory, fluid intelligence [18], and reasoning ability [19]. Individuals with higher working memory capacity are able to effectively adjust their attention to the requirements of the task, inhibit distracting stimuli, and flexibly use their cognitive resources (for a review in sport, see [20]). Given its impact on several mental processes, any approach aimed at enhancing working memory and other related executive functions (i.e., selective focused attention and cognitive flexibility) is particularly relevant in the athletic domain [21].

Together with psychological burdens, endocrine responses are reflective of race demands and competitive strain. Cortisol has been a widely used marker of stress. An elevation in the cortisol level, deriving from stimulation of the hypothalamic-pituitary-adrenal axis, indicates an individual’s experience of stress and/or physical effort [22]. Research in sport generally shows a negative relationship between cortisol and performance. Cortisol has also been found to influence decision making, attention, and memory by inhibiting information processing [23]. Another index of exercise intensity is chromogranin A, a soluble protein co-stored and co-released with catecholamines, deemed an accurate marker of the sympathetic adrenal activity [24–26]. Robazza et al. [27] assessed both salivary cortisol and chromogranin A of basketball players within an hour prior to games played at the team’s home venue across a whole season. Although the two biological markers were not related to performance, their salivary concentration was associated with perceived intensity, frequency, and functional impact of a number of psychobiosocial states. In line with the IZOF model assumptions [2], higher scores of functional states were linked to higher individual performance ratings.

Most research in sport has focused, so far, on assessment of hormone levels prior to or after competition [28–30]. Lautenbach, Laborde, Klämpfl, and Achtzehn [31] were the first who assessed the dynamics in cortisol levels, anxiety, affect intensity and valence, and performance parameters of two tennis players before, during, and after a match. Cortisol was negatively correlated with some performance parameters (e.g., unforced errors and return performance) and uncorrelated with other parameters (e.g., serving performance). These results, however, cannot be generalized because of the single subject nature of the study. Moreover, executive functions were not assessed.

### Study purpose and hypotheses

To date, no study has investigated the relationships among salivary cortisol, chromogranin A levels, psychobiosocial states, executive functions, and performance, and their fluctuations in pressurized contexts eliciting different levels of challenge and threat. Thus, drawing on the assumptions from the IZOF [2] and biopsychosocial [5] models, the purpose of this study was to examine the relationships among the variable levels and their changes over the course of meaningful competitive situations. A main contribution to the extant literature is that this study combined the IZOF [2] and biopsychosocial [5] theoretical frameworks in a single investigation. Findings were expected to provide support for the joint use of the two perspectives for both theoretical and applied objectives. From a conceptual standpoint, we predicted emotional, cognitive, psychophysiological, and performance variables to be related and to jointly change across different levels of cognitive and physical load. From a practical point of view, we explored the feasibility of implementing cognitive tasks to enhance the cognitive load for training purposes. Specific hypotheses were then formulated.

Regarding the relationships among variables, according to the IZOF and biopsychosocial models we hypothesized (H_1_) cortisol elevation responses under competitive pressure to be: (1) reflected in higher perceived exertion, (2) negatively related to functional psychobiosocial states and executive functions, and (3) positively related to dysfunctional psychobiosocial states. We did not formulate detailed predictions regarding chromogranin A relationships with the other variables due to the novel use of this marker in sport. However, previous research results on basketball players showed salivary concentration of chromogranin A associated with perceived beneficial effects of functional psychobiosocial states toward performance [27]. Based on these findings, we expected to find higher levels of chromogranin A related to better performance.

Concerning variable level fluctuations, we expected to find (H_2_) within-competition variations of variable scores in function of the different physical and psychological demands of the race. According to the biopsychosocial model, high levels of effort and skill requirements are antecedents of threat states [5]. Thus, we hypothesized that more physically and cognitively difficult routes would engender in orienteers higher perceived exertion, enhanced salivary cortisol levels, and related changes of all other variable scores (as stated in H_1_).

To further investigate the changes in the variable scores across competitive situations, we compared the orienteers’ psychophysiological responses in the usual condition of both physical and cognitive load with a condition in which the cognitive load was considerably reduced. To this purpose, the participants were asked to complete again the same course on the following day. The second race was therefore less psychologically demanding, because participants were acquainted with the course and did not need to use the map and compass. Lower levels of effort and skill requirements are likely conducive to challenge states [5]. Thus, we expected to find (H_3_) in the orienteers lower cortisol levels, higher levels of functional states, enhanced executive functions, lower levels of dysfunctional states, and improved performance.

## Method

### Participants

The sample consisted of 14 junior orienteering athletes, 7 boys and 7 girls, ranging in age from 15 to 20 years (*M* = 16.93 yrs., *SD* = 1.77). All participants were part of the Italian Junior National Team. Seven of them were skilled runners, with several years of practice (*M* = 5.85 yrs., *SD* = 1.35) and substantial amount of training during the week (*M* = 7.28 hrs., *SD* = 2.06). The other seven were medium level runners, with an average of four years of practice (*M* = 3.71 yrs., *SD* = 1.80) and a moderate amount of training per week (*M* = 5.57 hrs., *SD* = 1.57).

### Measures

#### Perceived exertion

Perceived exertion was rated on a modified Borg’s Category Ratio scale (CR-10 [32]) using the following verbal anchors: 0 = *nothing at all*, 0.5 = *very*, *very little*, 1 = *very little*, 2 = *little*, 3 = *moderately*, 5 = *much*, 7 = *very much*, 10 = *very*, *very much*, • = *maximal possible* (no verbal anchors were used for 4, 6, 8, and 9). The score of 11 is assigned to *maximal possible*. The CR-10 Borg scale has been shown to be closely related to various physiological and psychophysiological measurements in sport and exercise psychology [33, 34], and has been widely used to monitor training load [35].

#### Psychobiosocial states

Assessment was conducted using the psychobiosocial states scale, trait version (PBS-ST [3]). The scale is composed of 15 items, 8 functional and 7 dysfunctional, intended to assess seven modalities of a performance-related state (i.e., affective, cognitive, motivational, volitional, bodily-somatic, motor-behavioral, and operational). The scale derived from the original English version of the Individualized Profiling of Psychobiosocial States [4], and was validated to Italian language. Each item includes 3–4 descriptors conveying a similar experience that are categorized as functional or dysfunctional for performance. The aim is to transmit to the participants a straightforward depiction of an emotional experience. Specifically, the affective modality is assessed by three rows of synonym adjectives for: functional pleasant states, ‘enthusiastic, confident, carefree, joyful’; dysfunctional anxiety, ‘worried, apprehensive, concerned, troubled’; and functional anger, ‘fighting spirit, fierce, aggressive’. For the other six modalities, two rows of adjectives assess functional (+) or dysfunctional (-) states: Cognitive (+) modality, ‘alert, focused, attentive’; Cognitive (-), ‘distracted, overloaded, doubtful, confused’; Motivational (+), ‘motivated, committed, inspired’; Motivational (-), ‘unmotivated, uninterested, uncommitted’; Volitional (+), ‘purposeful, determined, persistent, decisive’; Volitional (-), ‘unwilling, undetermined, indecisive’; Bodily-somatic (+), ‘vigorous, energetic, physically-charged’; Bodily-somatic (-), ‘physically-tense, jittery, tired, exhausted’; Motor-behavioral (+), ‘relaxed-, coordinated-, powerful-, effortless-movement’; Motor-behavioral (-), ‘sluggish, clumsy, uncoordinated, powerless-movement’; Operational (+), ‘effective-, skillful-, reliable-, consistent-task execution’; and Operational (-), ‘ineffective-, unskillful-, unreliable-, inconsistent-task execution’.

The stem of items of the trait version was modified from ‘how do you usually feel’ to ‘how do you feel right now–at this moment’ in order to assess the current psychobiological states of participants. For each item of the scale athletes were requested to select one or more descriptors that best reflected their current state, and to rate the intensity on a 5-point Likert scale ranging from 0 (*not at all*) to 4 (*very*, *very much*). Mean scores of functional and dysfunctional items were computed. Robazza et al. [3] showed a two-factor solution (i.e., functional and dysfunctional intensity subscales) to be acceptable, with CFI = .950, TLI = .942, RMSEA (90% CI) = .108 (.098 ± .118), and SRMR = .121 in a sample of male and female athletes from different sports.

#### Memory

Three technical elements (symbols) were placed on each control point of the course, for a total of 48 elements (i.e., 12 symbols on the 4 control points comprised in a loop). The symbols were selected by three expert orienteering coaches and were customarily used in orienteering maps, such as trees, pits, marshes, springs, ponds, rock pillars, cliffs, caves, rocks, boulders, buildings, and fences. Orienteers were asked to memorize the 12 symbols they encountered on the four control points of a loop, and report them to the examiner in the main checking point within 30 sec. We deemed this ecological assignment to be a representative task in the assessment of working memory in the context of orienteering. Participants, indeed, engaged in an elaboration process of selective attention and inhibition in their effort to hold information in mind and, at the same time, to keep symbols separate from those included in the map, thereby avoiding interference during recall. The score was the number of symbols correctly reported.

#### Visual attention

The Bells Test was used to assess visual attention [36]. The test was originally developed to identify visual inattention (neglect) associated with clinical manifestation of attentional deficits in space [37]. Seven lines of 35 target figures (bells) are presented in a 21.5 × 28 cm sheet of paper interspersed with distractor figures (e.g., horse, bird, key, apple, mushroom, guitar, and car) in a pseudo-random manner. Each line contains 5 bells and 40 distractors. The paper was rotated 45° clockwise at the end of each loop to prevent habituation. Orienteers were required to circle with a pencil as many bells as possible in 30 sec. The score was the number of bells correctly circled. A number of validity studies documented the superiority of the Bells Test in detecting mild and moderate neglect, likely because this was a task demanding more selective attention in comparison with other measures [37].

#### Attention/Mental flexibility

We used the Trail Making Test as a measure of attention, speed, and mental flexibility [38]. Using a pencil, participants were required to connect 13 encircled numbers and 12 encircled letters randomly arranged on a page. The task consisted of connecting in 30 sec all letters and numbers in alternating order (i.e., 1, A, 2, B, 3, C, and so on). A normal printed version was administered after the first and third loops, while a specular version was used after the second and fourth loops to counteract habituation. The score was expressed in terms of the time in seconds required for task completion. Substantial research evidence has indicated the Trail Making Test to be a reliable and valid measure of attentional abilities, including visual search and visual-spatial sequencing, as well as speed and mental flexibility [38].

#### Cortisol and chromogranin A

Salivary cortisol and chromogranin A were obtained from saliva samples. The athletes were requested to refrain from ingesting stimulating (e.g., coffee and chocolate) or dye containing substances, and from brushing their teeth during the three hours before assessment. Saliva samples were collected by chewing regular cotton salivette sampling devices (Sarstedt, Nümbrecht, Germany), thus without chemical stimulants. Samples were kept on ice and then stored at -20°C until the day of analysis. At the day of the analysis, saliva samples were centrifuged 10 min at 2000 x g to remove particulate material. Hormonal determinations were obtained by using the Human Chromogranin A ELISA Kit (MyBiosource, San Diego, CA, USA) and Human Cortisol ELISA Kit (Diagnostics Biochem Canada Inc, London, Ontario, Canada) according to the manufacturers’ directions and were expressed as ng/ml. All samples were processed in duplicate during the same assay section.

#### Performance

The time to complete each loop was recorded in sec through Sport Ident Technology, an orienteering race timing system. This system consisted of a Sport Ident Card, an extended data memory stick fixed on an athlete’s finger, and a Sport Ident Station, an electronic device placed on each control point. The running time is automatically calculated by punching the Sport Ident Card into the Sport Ident Stations at each checking point. The intermediate time between control points of each loop, the total time of each loop, and the total time of the orienteering performance (four loops) were obtained for each participant.

### Procedure

The ethics committee for biomedical research of the “G. d’Annunzio” University of Chieti-Pescara, Italy, approved the study, with anonymity and confidentiality being assured for all the participants. A regional delegate of the Italian Federation of Orienteering and coaches of junior orienteers were initially contacted and informed about the study purposes. They showed interest in the investigation and agreed to organize a competition in a route located in a large natural area in the center of Italy. The athletes and their parents or guardians signed an informed consent form in accordance with the Declaration of Helsinki.

#### Day 1: Briefing

Participants were gathered nearby the competition site one the day before the commencement of the study. Upon their arrival, the orienteers were explained the main purposes of the study and procedures during a two-hour session. To create an experience of competing under pressure, we emphasized that the two-day competition was important, the race would be objectively assessed, and the final performance ranking would be evaluated by the coaches of the junior national team. During the initial two-hour session, all psychological measures were presented to help the participants become acquainted with the assessment procedures. Participants were recommended to abstain from consuming stimulating (e.g., coffee and chocolate) or dye containing substances, and to not brush their teeth three hours before collection of the saliva samples.

#### Day 2: First competitive race

The orienteering courses are usually composed of start and finish points, and a series of control points. The proposed course was comprised of four laps of different physical and psychological demands. To facilitate data collection, the course had a single start and finish point. Each lap included four control points. The orienteer’s task was to complete all laps in the shortest time possible passing through all control points with the aid of a map and a compass. Participants started the race at 9:00 a.m., three minutes apart.

**Fig 1:**
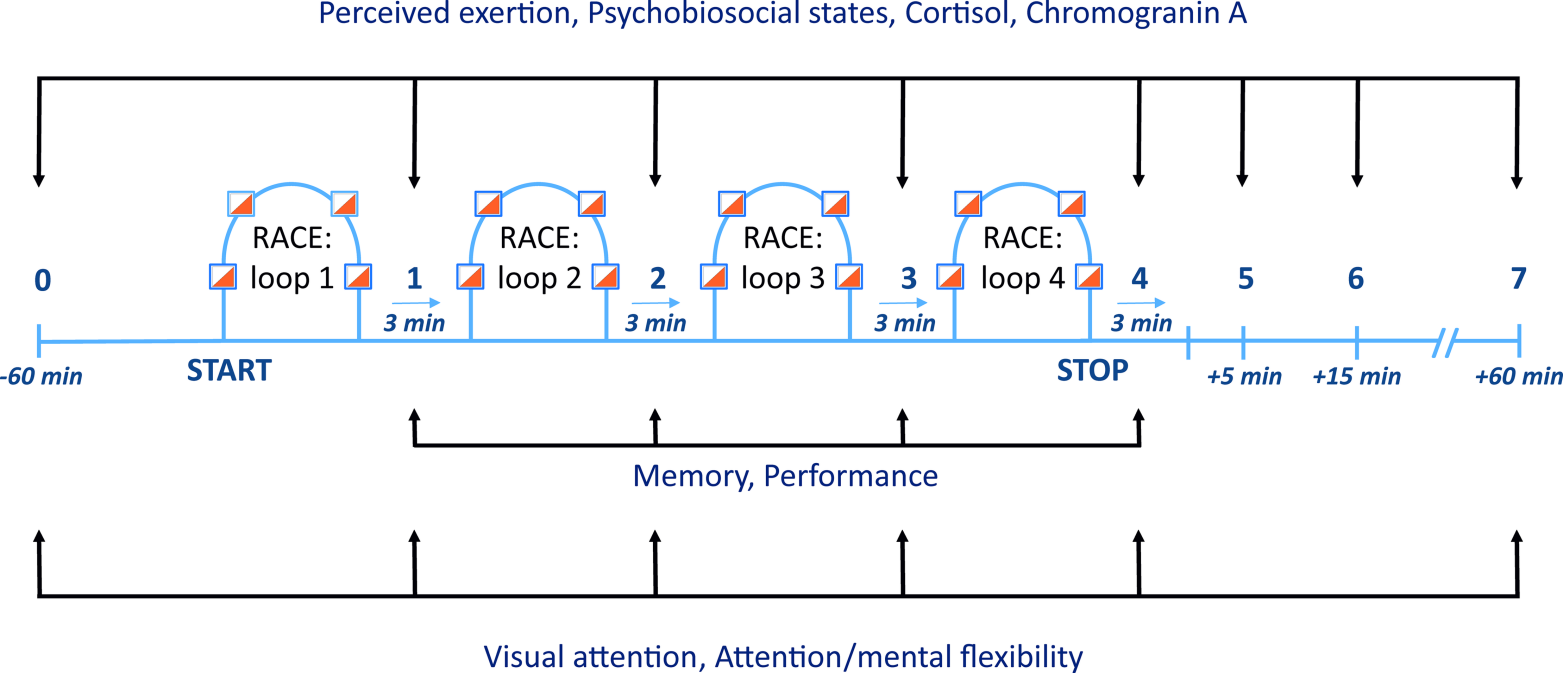
Timeline of assessment schedule across the investigation.

The assessment schedule is depicted in Fig 1. Eight assessments were performed to measure perceived exertion and psychobiosocial states, and to collect salivary samples. Specifically, such data were collected 60 min before the race, within 3 min after each loop, 5 min, 15 min, and 60 min after the race. Four memory assessments were carried out, one after each loop. Visual attention and attention/mental flexibility tests were administered at six different times (i.e., before the race start, after each loop, and 60 min after the race). The order of administration of the assessments after every loop was the following: (1) perceived exertion, (2) memory, (3) biomarkers, (4) visual attention, (5) attention/mental flexibility, and (6) psychobiosocial states. Six research assistants, with a specific measurement task each, were involved in the assessments. An additional experimenter supervised the whole procedure to ensure a correct measurement sequence and administration in a timely fashion.

Two hours after the race, all participants were allowed to walk across the same course led by a coach to memorize the specific features of the terrain (e.g., ground, environment) in preparation for the next race on the following day.

#### Day 3: Second competitive race

The second race took place on the same course, the following day. Participants started again the race at 9:00 a.m., three minutes apart, in the same order as in the first competition. Data were collected using the same administration procedures and participants’ starting order. Considering that the participants were already familiar with the course, they did not need to use a map and a compass. Thus, the cognitive load was largely reduced compared to the previous day, while the physical load remained about the same.

### Data analysis

Data were initially screened for missing cases, outliers, normality (using Shapiro–Wilk statistic), and sphericity [39]. A series of two–way repeated measures ANOVAs was then performed on the dependent variables. The independent variables were the competition day (two days) and the assessment phase (eight phases, from 0 to 7; see Fig 1). In particular, 2 × 4 (day × assessment phase) analysis was conducted on performance and memory data, 2 × 6 on visual attention and attention/mental flexibility data, and 2 × 8 on perceived exertion, cortisol, chromogranin A, and functional/dysfunctional psychobiosocial states. The sources of significant effects were then identified through pair–wise comparison of means.

## Results

**Fig 2:**
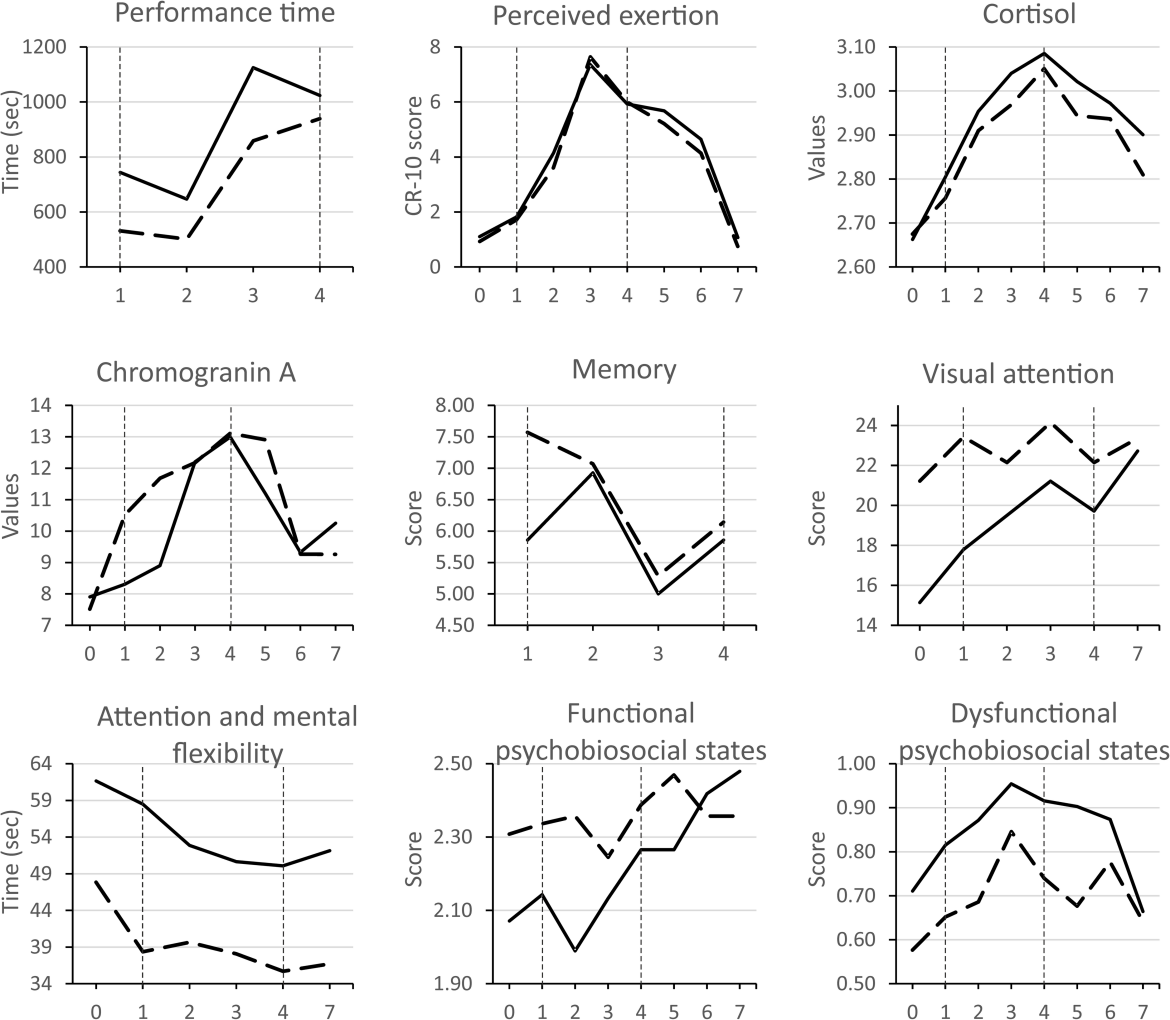
Trend over time of mean variable scores. Solid lines represent the data on the first competitive race, while dashed lines represent the data on the second competitive race. The numbers on the horizontal axis indicate the assessment phase: 0 = 60 min before the race (salivary samples) or just before the race (visual attention and attention/mental flexibility tests); 1 to 4 = after each loop; 5 to 7 = 5 min, 15 min, and 60 min after the race. The first loop and the fourth loop are also marked by vertical-dotted lines. Cortisol and chromogranin A are expressed as ng/ml.

Descriptive statistics and bivariate correlations among the variable scores collected across the two-day competition are reported in Table 1. The complete trend over time of the variable mean scores is shown in Fig 2. Evidence of non-normality was found for chromogranin A and dysfunctional psychobiosocial states. Thus, the data of these variables were transformed using square root transformation before conducting the main analysis [40]. Across the two-day competition, low to moderate negative correlations were shown between performance time and chromogranin A, visual attention, and functional psychobiosocial states, while moderate positive correlations were observed between performance time and dysfunctional psychobiosocial states. Furthermore, cortisol levels were negatively related to memory, visual attention, and functional psychobiosocial states, and positively related to dysfunctional psychobiosocial states.

**Table 1:** Descriptive statistics and pearson correlation coefficients of mean scores of measures collected across the four loops of the orienteering course.

Measures	*M*	*SD*	1	2	3	4	5	6	7	8
**Day 1**										
**1. Performance time (in sec)**	884.82	227.59	—							
**2. Perceived exertion**	5.90	1.54	.02	—						
**3. Cortisol**	2.97	0.13	.15	.32^†^	—					
**4. Chromogranin A**	10.60	5.79	-.22^†^	-.10	-.34^†^	—				
**5. Memory**	5.91	1.63	-.24^†^	.11	-.27^†^	-.27^†^	—			
**6. Visual attention**	19.55	3.21	-.39^†^	.13	-.56^††^	.28^†^	.35^†^	—		
**7. Attention/mental flexibility**	53.02	8.27	.45^††^	-.16	.16	.12	-.73^†††^	-.49^††^	—	
**8. Functional psychobiosocial states**	2.27	0.61	-.30^†^	.07	-.36^†^	.19	.15	.20	.13	—
**9. Dysfunctional psychobiosocial states**	0.91	0.37	.48^††^	.43^††^	.59^††^	-.04	-.14	-.19	-.04	-.56^††^
**Day 2**										
**1. Performance time**	707.77	159.88	—							
**2. Perceived exertion**	5.75	1.03	.19	—						
**3. Cortisol**	2.92	0.16	-.02	.46^††^	—					
**4. Chromogranin A**	11.87	6.61	-.42^††^	-.16	-.09	—				
**5. Memory**	6.52	1.46	.21^†^	-.48^††^	-.35^†^	.27^†^	—			
**6. Visual attention**	22.96	3.15	-.38^†^	.06	-.35^†^	.20^†^	.26^†^	—		
**7. Attention/mental flexibility**	37.95	6.50	.14	.26^†^	.26^†^	-.24^†^	-.44^††^	-.66^†††^	—	
**8. Functional psychobiosocial states**	2.36	0.80	-.29^†^	.15	-.33^†^	-.15	-.34^†^	.11	.45^††^	—
**9. Dysfunctional psychobiosocial states**	0.76	0.36	.53^††^	.38^†^	.55^††^	.00	.04	-.23^†^	-.14	-.72^†††^

*Note*. Scores of chromogranin A and dysfunctional psychobiosocial states are normalized using square root transformation.

^†^Low correlation.

^††^Moderate correlation.

^†††^Moderately high correlation.

ANOVA results are contained in Table 2. Sphericity assumptions were examined through the Mauchly’s test and, in case of violation, Greenhouse–Geisser correction in the degrees of freedom was applied. Compared to the first race, better performance time, visual attention scores, and attention/mental flexibility scores were reported on the second race. Lower levels of cortisol and dysfunctional psychobiosocial states were also found. With the exception of functional psychobiosocial states, significant differences were observed on the scores of all variables across the assessment phases. Pair–wise comparisons showed significant differences (*p* < .050) in a number of variable scores across assessment phases (see S1 Data set and post-hoc test results). Interestingly, performance time (*p* < .001), perceived exertion (*p* < .005), cortisol levels (*p* < .004), and chromogranin A levels (*p* < .050) of the two races were higher on loops 3 and 4 compared to loops 1 and 2. Dysfunctional psychobiosocial states scores (*p* < .009) were larger on loop 3 compared to loops 1 and 2, while memory scores were lower (*p* < .005). Mean performance time of the race (*p* < .001), and mean performance scores of visual attention (*p* < .001) and of attention/mental flexibility (*p* < .001) improved from day 1 to day 2, while mean intensity scores of dysfunctional psychobiosocial states decreased (*p* < .050). However, post-hoc results should be interpreted with caution because of the small sample size and the number of comparisons.

**Table 2:** Analysis of variance results.

Measure	Source	*F*	*df*	*p* value	ƞ_p_^2^	Power
**Performance time**	Day	22.85	1, 13	< .001	.64	.99
	Assessment	43.69	3, 39	< .001	.77	1.00
	Day × Assessment	1.47	3, 39	.239	.10	.36
**Perceived exertion**	Day	0.67	1, 13	.429	.05	.12
	Assessment	70.40	2.074, 26.968	< .001	.84	1.00
	Day × Assessment	0.53	2.899, 37.681	.658	.04	.15
**Cortisol**	Day	9.43	1, 13	.009	.42	.81
	Assessment	32.06	2.661, 34.588	< .001	.71	1.00
	Day × Assessment	1.03	3.139, 40.801	.392	.07	.26
**Chromogranin A**	Day	0.70	1, 13	.418	.05	.12
	Assessment	3.41	3.201, 41.609	.024	.21	.75
	Day × Assessment	1.07	7, 91	.390	.08	.44
**Memory**	Day	2.67	1, 13	.126	.17	.33
	Assessment	7.03	3, 39	.001	.35	.97
	Day × Assessment	1.97	3, 39	.134	.13	.47
**Visual attention**	Day	38.07	1, 13	< .001	.75	1.00
	Assessment	9.57	5, 65	< .001	.42	1.00
	Day × Assessment	4.84	5, 65	.001	.27	.97
**Attention/mental flexibility**	Day	221.04	1, 13	< .001	.94	1.00
	Assessment	9.55	5, 65	< .001	.42	1.00
	Day × Assessment	1.64	5, 65	.163	.11	.53
**Functional psychobiosocial states**	Day	0.78	1, 13	.392	.06	.13
	Assessment	1.39	2.677, 34.806	.264	.10	.32
	Day × Assessment	1.62	7, 91	.139	.11	.64
**Dysfunctional psychobiosocial states**	Day	4.94	1, 13	.045	.28	.54
	Assessment	3.85	7, 91	.001	.23	.97
	Day × Assessment	0.36	3.142, 40.850	.789	.03	.12

## Discussion

The purpose of this study was to investigate the relationships among psychobiosocial states, cognitive executive functions, and endocrine responses of orienteers involved in a two-day competitive race based on the assumptions of the IZOF [2] and biopsychosocial [5] models. Combining predictions and indications stemming from both views can provide a better understanding of athletes’ psychophysiological reactions in pressurized contexts and also inform applied interventions.

Our findings provided support for the first hypothesis (H_1_), showing that an elevation in cortisol levels due to competitive pressure, and mirrored in higher perceived exertion, was associated with higher intensity of dysfunctional psychobiosocial states, lower intensity of functional psychobiosocial states, and decay in memory, visual attention, and attention/mental flexibility. Results are consistent with previous research on the negative influence of increased cortisol levels on cognitive processes, including working memory, attention control, and decision making [23]. The worsening in the athletes’ psychobiosocial states (i.e., higher intensity of dysfunctional states and lower intensity of functional states) and top-down executive functions may hamper performance. More importantly, our findings concur with a body of IZOF-based research evidence in sport generally suggesting that a high probability of optimal functioning can occur when the athlete experiences a combination of high intensity of functional psychobiosocial states and low intensity of dysfunctional psychobiosocial states [41]. Conversely, less than optimal functioning is associated with low levels of functional states and high levels of dysfunctional states. Results are also in line with the predictions stemming from the biopsychosocial model of challenge and threat [5, 13] and its application to the sport and performance contexts [12]. Using a golf putting task, for example, Moore et al. [10] manipulated the instructions provided to novice golfers to create a challenge group and a threat group. The challenge group executed more accurately, and showed more efficient putting kinematics and forearm muscle activity than the threat group. Similarly, in a pressurized environment requiring accurate execution of a novel motor task (i.e., laparoscopic surgery), Vine et al. [42] found that evaluating the task as challenging resulted in effective attentional control and superior performance. In our study, increased levels of cortisol and dysfunctional emotions and decreased cognitive processes (i.e., memory, visual attention, and attention/mental flexibility) suggest a state typified in terms of threat rather than challenge.

According to our second hypothesis (H_2_), we found within-competition fluctuations of psychophysiological variables. Specifically, the third and fourth race loops seemed to be more difficult than the first and second ones in terms of both physical and psychological demands. Increased difficulty requires more effort and skills, which can induce a threat state with related emotional and cognitive consequences. Indeed, race difficulty was manifested in slower performance times and higher levels of perceived exertion, cortisol, chromogranin A, and dysfunctional psychobiosocial states. Lower scores of memory and attention/mental flexibility were also observed (Table 2 and Fig 2). Modifications in psychophysiological responses can be interpreted again within the tenets of the IZOF model [2] and the biopsychosocial model of challenge and threat [5]. Both models, indeed, emphasize the interplay among emotional, cognitive, biological, and social modalities for an individual’s adaptation to environmental changes.

The second day we observed faster performance times, lower intensity of dysfunctional psychobiosocial states, and improved visual attention and attention/mental flexibility (Table 2 and Fig 2). These results highlight the impact of the cognitive burden usually associated with orienteering race that is added to the physical load [15]. Findings are also aligned with our last study hypothesis (H_3_) stating that a substantial reduction in the cognitive load of the race, due to the familiarity with the course, would result in enhanced psychophysical states and improved performance. As postulated in the biopsychosocial model, familiarity and low levels of effort and skill requirements can lead to a challenge state. When the cognitive load specific to the race was removed, more cognitive resources were available for other cognitive tasks. Notably, while the cortisol levels were lower across the second-day race compared to the first day, the athletes’ chromogranin A levels did not differ significantly in the two competitions. This finding, together with the correlation observed between chromogranin A and executive functions (even though small), may support the view of chromogranin A as a marker of the sympathetic adrenal activity in response to exercise intensity [24, 26]. Low and moderate correlations across the two races were also shown between chromogranin A levels and performance times suggesting that higher levels of chromogranin A were related to better performance. This is in accordance with previous IZOF-based research results in basketball players showing salivary concentration of chromogranin A to be associated with perceived beneficial effects of functional psychobiosocial states on performance [27].

Taken together, findings of the current study can be understood in light of the combined and unique contributions of the IZOF [2] and biopsychosocial [5] theoretical frameworks. Both models build upon the Lazarus’ [6] notion that the athlete’s appraisal of situational demands and personal resources determines the perception of a situation as challenging or threatening. Within this combined view, the contribution of the IZOF model is more on the description, prediction, explanation, and self-regulation of a wide range of functional or dysfunctional psychobiosocial experiences that accompany challenge or threat. In contrast, the biopsychosocial model focuses on the distinct patterns of neuroendocrine and cardiovascular activity of challenge and threat indexed objectively as well as subjectively [5, 14]. Drawing on both perspectives, results of this study highlighted the expected relationships among a number of psychological, physiological, and performance variables, as well as their fluctuations as a function of cognitive and physical demands. Variations in cognitive and physical loads within the race (different loops) and between races (day 1 and day 2) were likely conducive to changes in the individual experience along the challenge–threat continuum, also manifested in the observed psychophysiological responses.

Notwithstanding the encouraging findings, we acknowledge some study limitations. First, the low power associated with the small sample size reduces the generalizability of the results. However, our purposeful sample of high-medium competitive level athletes and the within-subjects design, in which participants served as their own controls, tend to enhance the power of the analysis [43]. Second, we did not assess the fluctuations of one’s appraisal of perceived competitive demands and personal coping resources to identify the individual’s state within the challenge–threat continuum postulated in the biopsychosocial model (e.g., [10]). Finally, the present study examined the effects of competitive pressure in high and medium level junior orienteers. Given the small sample size, potential differences by level, experience, and gender were not examined. Thus, future research should aim to investigate the psychophysiological effects of competitive pressure in larger samples, taking into account individual appraisals of situational demands and coping resources, as well as individual differences such as performance level, experience, age, and gender. Despite these issues, the current study had high ecological validity, and findings can be regarded as valuable preliminary evidence to promote further research for a better understanding of the athletes’ experience during competition.

From a practical perspective, this study provides novel findings that may inform strategies practitioners apply to assist orienteers in dealing with performance and competitive demands. In particular, helping athletes become aware of their performance-related psychobiosocial states and their effects on performance, cognitive functions, and endocrine responses can be an important step toward self-regulation of thoughts, feelings, attention focus, and behaviors to achieve performance goals. The procedure adopted in the current study also suggests the feasibility of including additional cognitive load to usual performance, with the purpose to deplete and then replenish and increase cognitive resources through training. According to the strength model of self-control [44, 45], for example, it might be speculated that the working memory task implemented during the route (i.e., recalling the symbols placed on the control points of the course) may be used to strengthen working memory, which is a critical ability in orienteering. Future research should examine the effects of an increase in cognitive load during training on individual’s resources and performance in orienteering and other sports.

In conclusion, this investigation provides a unique contribution to the literature on psychobiosocial states, cognitive functions, endocrine responses, and performance under competitive pressure. To our knowledge, this is the first study that combines the IZOF [2] and biopsychosocial [5] theoretical views. Findings offer initial and substantial support for the joint use of the two perspectives for both theoretical and practical purposes. Theoretically, the pattern of results highlight the expected relationship among emotional, cognitive, psychophysiological, and performance variables, as well as their changes across different levels of cognitive and physical load. From a practical perspective, findings support the feasibility of implementing training tasks to increase cognitive resources, and suggest potential benefits derived from the self-regulation of psychobiosocial states to deal with performance and competitive demands. Future research combining the IZOF and biopsychosocial theoretical frameworks is warranted.

## Supporting information

S1 Data set and post-hoc test results (PDF)
