# Supplementary material for: Psychophysiological responses of junior orienteers under competitive pressure
Source: PLoS One. 2018 Apr 26;13(4):e0196273. doi: 10.1371/journal.pone.0196273 (PMC5919653; doi:10.1371/journal.pone.0196273)
Supplement: S1 Data set and post-hoc test results — (PDF) [file pone.0196273.s001.pdf]

S1 Data set and post-hoc test results

Day 1

	Performance time					Perceived exertion							
**Participant**	P1	P2	P3	P4		PE0	PE1	PE2	PE3	PE4	PE5	PE6	PE7
1	629	641	1727	1148		0.5	2	3	5	3	5	4	0
2	544	537	1364	935		0.5	0.5	0.5	5	3	4	3	0
3	917	615	1158	1178		1	3	3	5	5	5.5	6	5
4	720	834	1131	1185		3	5	8	8	6	7	6	4
5	806	1109	1648	1163		3	0.5	7	7	5	5	1	0
6	735	978	860	925		1	3	8	10	9	8	5	0
7	1081	467	1644	1139		0.5	2	7	8	7	7	7	0
8	693	479	884	913		0	0	0.5	10	8	7	6	0
9	440	407	679	701		0.5	1	3	6	4	3	3	2
10	736	695	1096	1587		2	3	5	7	7	5	4	2
11	465	353	778	871		0.5	1	2	8	6	5	3	0.5
12	529	354	664	667		1	1	3	10	8	8	6	1
13	1573	854	1427	1162		2	3	7	8	7	6	7	0.5
14	549	730	686	760		0	0.5	1	6	5	4	4	0

Day 2

1	596	589	1051	1079		0.5	1	3	7	5	6	5	0
2	468	466	790	894		1	2	7	6	5	4	4	0.5
3	548	592	1125	1539		3	4	6	9	6	5	4	1
4	582	614	998	1210		1	3	4	6	5	4	5	1
5	517	455	781	1075		0.5	2	3	6	4	4	3	2
6	423	511	619	770		0	0.5	2	10	9	7	5	0
7	662	608	967	966		0	1	3	9	7	4	3	0
8	613	462	849	926		0	0	0.5	8	7	7	5	0
9	376	325	595	607		0.5	1	4	8	4	4	2	0.5
10	841	546	1009	1097		2	3	6	7	6	5	5	3
11	387	409	586	659		0.5	0.5	3	7	6	5	3	0.5
12	465	327	840	601		1	1	2	9	7	6	4	1
13	561	797	1073	1115		3	4	6	8	8	7	6	1
14	404	316	743	611		0	1	1	7	5	5	4	0

Day 1

Cortisol

Participant	C0	C1	C2	C3	C4	C5	C6	C7
1	2.738	2.760	2.965	3.206	3.207	3.107	2.939	2.906
2	2.481	2.690	2.742	2.924	3.041	2.850	2.735	2.807
3	2.446	2.814	2.891	3.053	2.963	2.850	2.857	2.598
4	2.276	2.404	2.712	2.907	2.958	2.927	2.835	2.634
5	2.300	2.649	2.998	3.126	3.051	3.057	3.025	2.840
6	2.989	3.099	3.109	3.189	3.262	3.189	3.210	3.160
7	3.086	3.021	3.085	3.154	3.160	3.132	3.168	3.108
8	3.008	3.077	3.162	3.222	3.255	3.179	3.182	3.169
9	2.958	3.005	3.005	2.947	2.991	3.144	2.992	3.159
10	3.017	3.054	3.072	3.056	3.207	3.116	3.111	3.089
11	2.496	2.763	2.902	2.923	3.078	2.945	2.933	2.855
12	2.406	2.633	2.779	2.993	3.029	2.869	2.904	2.682
13	2.504	2.635	3.067	2.985	3.034	3.086	2.922	2.884
14	2.568	2.656	2.861	2.882	2.957	2.842	2.797	2.721

Day 2

1	2.612	2.720	2.967	3.004	3.143	2.782	2.832	2.779
2	2.624	2.773	2.787	2.833	2.945	2.804	2.797	2.687
3	2.540	2.640	2.835	2.970	2.976	2.857	2.818	2.614
4	2.387	2.341	2.662	2.690	2.861	2.771	2.711	2.568
5	2.703	2.626	2.584	2.774	2.850	2.812	2.899	2.817
6	2.995	3.047	3.169	3.128	3.176	3.094	3.186	3.119
7	2.961	2.995	3.123	3.034	3.124	2.906	3.064	3.012
8	2.959	2.953	3.033	3.190	3.173	3.208	3.189	3.213
9	2.886	2.897	3.089	2.966	3.138	3.058	3.010	3.103
10	2.979	3.121	3.148	3.226	3.254	3.229	3.231	3.113
11	2.461	2.679	2.900	3.052	3.132	3.012	2.829	2.743
12	2.336	2.636	2.791	2.883	3.030	2.864	2.847	2.823
13	2.521	2.539	2.950	3.029	3.009	3.010	2.916	2.727
14	2.481	2.631	2.700	2.789	2.900	2.805	2.783	2.014

Day 1

Chromogranin A

Participant	CgA0	CgA1	CgA2	CgA3	CgA4	CgA5	CgA6	CgA7
1	4.671	11.166	14.992	204.306	78.050	8.797	3.914	43.287
2	3.515	4.357	6.384	5.442	4.400	5.684	27.355	4.086
3	19.318	8.254	22.145	157.139	135.482	186.204	140.450	70.269
4	326.695	322.969	256.014	327.780	444.586	356.032	332.962	297.086
5	79.720	430.510	418.475	412.865	372.107	424.100	109.699	273.245
6	11.009	146.646	78.207	55.493	270.090	17.091	26.156	9.853
7	7.926	5.256	5.313	6.570	5.570	6.541	7.355	7.969
8	9.296	18.219	35.264	142.977	67.514	64.216	47.542	270.761
9	326.695	386.982	386.982	336.959	244.779	43.330	35.750	314.546
10	11.009	4.257	18.918	13.122	151.028	5.270	9.182	18.047
11	30.882	76.665	309.849	334.489	393.463	380.701	344.625	13.037
12	381.200	38.948	19.689	357.474	384.327	247.962	344.211	410.238
13	5.113	123.447	33.894	19.004	225.049	352.149	74.609	36.306
14	299.385	7.683	65.201	315.731	92.111	429.625	175.083	341.385

Day 2

1	11.024	10.624	4.143	7.041	10.353	49.398	12.094	253.659
2	3.757	5.470	4.143	4.185	7.069	4.285	4.114	7.812
3	32.823	32.138	281.211	108.472	222.951	309.192	85.373	153.298
4	170.187	284.809	311.034	325.281	352.377	334.875	220.324	200.837
5	32.095	317.872	374.533	406.769	417.547	438.390	217.697	59.762
6	81.833	308.536	359.187	390.580	281.097	149.858	320.213	6.784
7	4.014	5.770	4.185	9.667	15.963	17.862	4.228	6.912
8	107.658	214.028	152.185	97.965	118.793	129.186	140.735	189.088
9	406.455	362.770	430.710	285.037	392.022	312.704	312.090	410.152
10	7.912	22.958	76.907	305.666	300.712	17.805	5.185	23.372
11	21.559	398.874	356.831	387.724	390.209	458.819	7.840	13.351
12	227.848	21.688	194.784	367.852	381.686	240.153	331.220	45.300
13	3.929	3.543	20.803	32.266	36.107	197.368	15.178	7.026
14	88.657	265.322	29.782	29.854	89.813	197.725	75.680	381.557

Day 1

	Memory					Visual attention					
**Participant**	M1	M2	M3	M4		VA0	VA1	VA2	VA3	VA4	VA7
1	8	10	3	4		11	18	14	22	16	25
2	7	8	4	8		13	20	18	22	18	20
3	10	10	9	8		17	26	24	21	22	26
4	7	10	7	7		11	17	22	29	22	24
5	3	4	1	2		15	18	17	19	17	27
6	6	5	4	4		11	15	21	19	20	21
7	7	6	2	4		14	18	24	22	21	20
8	6	7	6	8		11	14	14	19	15	17
9	4	7	4	7		20	17	19	20	23	20
10	3	5	7	9		17	10	13	17	14	20
11	6	5	5	4		24	22	24	23	25	20
12	6	7	7	9		16	21	26	26	26	34
13	4	8	5	3		15	12	20	17	21	22
14	5	5	6	5		17	21	17	21	16	22

Day 2

1	7	8	5	7		19	25	24	28	17	24
2	9	7	5	7		20	24	24	23	24	22
3	11	11	4	8		25	26	23	28	22	27
4	10	9	9	8		26	23	26	31	24	29
5	10	5	5	5		14	23	16	18	18	19
6	5	6	2	5		20	26	24	24	22	25
7	4	4	4	6		23	22	16	25	22	21
8	7	5	6	5		18	21	18	22	24	25
9	9	9	7	10		22	24	22	24	22	22
10	8	7	6	7		20	15	22	13	20	18
11	7	6	6	4		26	23	27	27	25	22
12	6	7	7	7		29	31	27	30	30	31
13	7	8	5	2		17	20	19	21	18	18
14	6	7	3	5		18	25	22	24	22	24

Day 1

	Attention/mental flexibility							Functional psychobiosocial states					
**Participant**	AMF0	AMF1	AMF2	AMF3	AMF4	AMF7		FPBSS0	FPBSS1	FPBSS2	FPBSS3	FPBSS4	FPBSS5
1	61	58	50	44	55	44		1.57	1.57	1.14	0.86	1.00	2.00
2	60	55	54	56	38	41		1.00	1.14	1.57	3.57	3.14	2.71
3	65	42	38	45	42	39		2.14	2.43	2.14	2.14	2.14	2.43
4	61	61	49	36	62	58		2.57	2.71	2.71	2.57	3.14	2.43
5	70	65	65	52	65	60		1.29	2.00	1.43	1.86	2.29	1.43
6	60	70	64	54	61	47		2.43	2.43	2.29	2.29	2.71	2.29
7	63	55	60	60	49	53		1.86	1.71	1.14	1.14	1.00	1.71
8	55	59	49	44	37	64		2.00	2.14	2.14	2.43	2.71	3.00
9	60	58	50	59	58	48		2.29	2.43	2.43	2.86	2.29	3.14
10	63	60	49	52	47	62		1.14	1.43	0.86	1.00	0.86	0.86
11	63	60	48	44	42	53		2.14	1.71	1.86	1.71	2.14	2.43
12	49	43	37	32	30	30		2.29	2.57	2.43	2.14	2.14	2.14
13	67	69	63	65	61	58		3.29	3.57	3.14	3.14	2.86	3.29
14	66	64	64	66	54	73		3.00	2.14	2.57	2.14	3.29	1.86

Day 2

1	44	37	40	40	35	32		1.71	2.00	2.00	2.00	2.14	2.14
2	43	32	36	36	31	26		0.86	1.14	1.71	2.29	2.29	2.00
3	35	32	54	26	33	28		2.29	2.14	1.43	1.43	1.71	2.29
4	36	34	37	27	34	30		2.57	2.86	2.71	2.43	2.57	2.57
5	60	36	45	38	37	44		1.29	1.71	1.71	1.86	2.14	2.29
6	50	44	45	47	45	38		3.29	3.29	3.00	2.71	2.43	2.86
7	39	38	37	48	35	36		2.57	3.14	3.14	3.14	2.71	2.00
8	39	52	38	40	31	64		2.60	2.14	2.43	2.14	2.86	2.71
9	50	44	32	38	36	33		2.86	2.57	2.71	2.71	2.86	2.43
10	50	44	41	37	44	30		0.71	0.29	0.57	0.43	0.14	0.71
11	62	31	32	26	27	32		2.29	2.43	2.43	2.00	1.86	2.14
12	38	26	23	25	24	24		2.00	1.57	1.71	1.57	1.71	2.29
13	63	47	40	52	40	53		3.71	3.71	3.57	3.29	3.43	3.71
14	61	40	55	53	48	44		3.57	3.71	3.86	3.43	4.57	4.43

Day 1

Dysfunctional psychobiosocial states

FPBSS6	FPBSS7	**Participant**	DPBSS0	DPBSS1	DPBSS2	DPBSS3	DPBSS4	DPBSS5	DPBSS6	DPBSS7
2.00	2.14	1	0.333	0.500	0.667	1.000	1.333	0.500	0.167	0.000
2.29	1.71	2	0.167	0.167	0.333	0.167	0.167	0.000	0.167	0.167
2.14	2.43	3	0.833	1.000	1.167	1.667	2.000	1.833	1.667	1.500
2.43	2.57	4	0.833	1.000	0.667	0.667	0.667	0.333	0.333	0.667
1.86	2.29	5	1.667	1.500	2.333	1.833	1.000	2.167	2.167	0.500
2.43	3.43	6	1.000	1.333	1.833	1.167	1.333	2.333	2.333	0.000
2.29	2.71	7	0.667	1.167	1.333	2.167	1.167	1.667	0.667	0.000
3.14	3.43	8	0.333	0.667	0.500	1.000	1.000	1.167	1.333	0.833
2.71	2.71	9	0.167	0.000	0.000	0.167	0.167	0.000	0.833	0.333
1.43	1.71	10	2.000	2.333	1.833	2.167	1.833	1.833	1.500	1.167
2.86	2.57	11	0.667	1.000	1.167	1.833	1.000	0.333	0.500	0.500
2.29	2.43	12	0.167	0.833	0.667	1.000	0.500	0.500	0.667	1.000
3.00	2.57	13	0.167	0.333	0.833	0.500	0.833	0.833	0.833	0.500
3.00	2.00	14	0.000	0.000	0.000	0.000	0.167	1.167	0.000	1.333

Day 2

2.00	2.29	1	0.500	0.500	0.333	0.500	0.500	0.500	0.500	0.167
1.71	1.14	2	0.000	0.000	0.167	0.833	0.000	0.000	0.333	0.167
2.14	2.43	3	1.667	1.667	1.500	1.500	1.333	0.500	0.833	0.833
2.71	2.71	4	0.500	1.000	0.667	0.333	0.667	0.833	0.333	0.667
2.00	2.00	5	0.500	0.333	0.167	0.667	0.167	0.000	0.500	0.333
2.71	2.14	6	0.167	0.333	0.333	0.833	0.167	0.500	0.667	1.667
2.71	3.00	7	0.167	0.167	0.667	0.500	0.833	1.167	1.000	0.500
2.43	3.29	8	0.250	0.333	0.333	1.500	0.833	0.667	0.667	0.333
2.57	2.14	9	0.167	0.167	0.167	0.167	0.167	0.167	0.167	0.000
0.71	0.86	10	2.000	2.833	2.500	3.167	3.000	2.833	2.333	2.167
2.14	2.14	11	0.167	0.333	0.167	0.500	1.000	0.667	0.500	0.667
2.29	2.29	12	0.500	0.833	1.500	1.333	1.167	0.667	0.500	0.333
3.29	3.14	13	0.167	0.167	0.333	0.500	0.667	0.667	0.500	0.167
3.57	3.43	14	0.000	0.000	0.000	0.000	0.000	0.000	0.500	0.000

Pairwise comparisons

Performance time Day 1

(I) Loop	(J) Loop	Mean difference (I-J)	Std. Error	p level	95% CI	
					Lower limit	Higher limit
1	2	97.429	79.172	0.240	-73.612	268.469
	3	-380.643	90.711	0.001	-576.613	-184.673
	4	-279.786	74.702	0.002	-441.169	-118.402
2	3	-478.071	97.840	0.000	-689.442	-266.701
	4	-377.214	68.475	0.000	-525.146	-229.282
3	4	100.857	81.248	0.236	-74.669	276.383

Performance time Day 2

(I) Loop	(J) Loop	Mean difference (I-J)	Std. Error	p level	95% CI	
					Lower limit	Higher limit
1	2	30.429	33.145	0.375	-41.178	102.035
	3	-327.357	33.702	0.000	-400.167	-254.548
	4	-407.571	59.712	0.000	-536.572	-278.571
2	3	-357.786	32.706	0.000	-428.442	-287.129
	4	-438.000	52.061	0.000	-550.471	-325.529
3	4	-80.214	43.384	0.087	-173.939	13.511

Perceived exertion Day1

(I) Phase	(J) Phase	Mean difference (I-J)	Std. Error	p level	95% CI	
					Lower limit	Higher limit
1	2	-0.714	0.318	0.042	-1.400	-0.028
	3	-3.036	0.580	0.000	-4.289	-1.783
	4	-6.250	0.536	0.000	-7.409	-5.091
	5	-4.821	0.539	0.000	-5.985	-3.658
	6	-4.571	0.450	0.000	-5.545	-3.598
	7	-3.536	0.566	0.000	-4.758	-2.314
	8	0.036	0.421	0.934	-0.873	0.945
2	3	-2.321	0.556	0.001	-3.523	-1.120
	4	-5.536	0.606	0.000	-6.844	-4.228
	5	-4.107	0.558	0.000	-5.312	-2.902
	6	-3.857	0.449	0.000	-4.827	-2.887
	7	-2.821	0.447	0.000	-3.786	-1.857
	8	0.750	0.358	0.057	-0.024	1.524
3	4	-3.214	0.752	0.001	-4.838	-1.590
	5	-1.786	0.714	0.027	-3.329	-0.243
	6	-1.536	0.666	0.038	-2.975	-0.097
	7	-0.500	0.784	0.535	-2.195	1.195
	8	3.071	0.808	0.002	1.325	4.818
4	5	1.429	0.202	0.000	0.992	1.865
	6	1.679	0.285	0.000	1.062	2.295
	7	2.714	0.518	0.000	1.595	3.834
	8	6.286	0.728	0.000	4.714	7.858
5	6	0.250	0.291	0.405	-0.378	0.878
	7	1.286	0.462	0.016	0.287	2.284
	8	4.857	0.688	0.000	3.370	6.344
6	7	1.036	0.365	0.014	0.247	1.824
	8	4.607	0.600	0.000	3.310	5.904
7	8	3.571	0.559	0.000	2.363	4.780

Perceived exertion Day2

(I) Phase	(J) Phase	Mean difference (I-J)	Std. Error	p level	95% CI	
					Lower limit	Higher limit
1	2	-0.786	0.155	0.000	-1.120	-0.451
	3	-2.679	0.369	0.000	-3.476	-1.881
	4	-6.714	0.431	0.000	-7.646	-5.782
	5	-5.071	0.450	0.000	-6.045	-4.098
	6	-4.286	0.398	0.000	-5.146	-3.425
	7	-3.214	0.318	0.000	-3.900	-2.528
	8	0.179	0.249	0.486	-0.360	0.717
2	3	-1.893	0.336	0.000	-2.619	-1.166
	4	-5.929	0.532	0.000	-7.077	-4.780
	5	-4.286	0.541	0.000	-5.455	-3.116
	6	-3.500	0.508	0.000	-4.598	-2.402
	7	-2.429	0.370	0.000	-3.228	-1.629
	8	0.964	0.285	0.005	0.350	1.579
3	4	-4.036	0.700	0.000	-5.548	-2.523
	5	-2.393	0.707	0.005	-3.921	-0.864
	6	-1.607	0.704	0.040	-3.127	-0.087
	7	-0.536	0.580	0.373	-1.789	0.717
	8	2.857	0.467	0.000	1.848	3.866
4	5	1.643	0.269	0.000	1.061	2.225
	6	2.429	0.327	0.000	1.723	3.134
	7	3.500	0.442	0.000	2.546	4.454
	8	6.893	0.472	0.000	5.873	7.913
5	6	0.786	0.261	0.010	0.223	1.349
	7	1.857	0.345	0.000	1.111	2.603
	8	5.250	0.497	0.000	4.177	6.323
6	7	1.071	0.245	0.001	0.542	1.601
	8	4.464	0.437	0.000	3.521	5.408
7	8	3.393	0.360	0.000	2.615	4.171

Cortisol Day 1

(I) Phase	(J) Phase	Mean difference (I-J)	Std. Error	p level	95% CI	
					Lower limit	Higher limit
1	2	-0.142	0.034	0.001	-0.215	-0.069
	3	-0.291	0.055	0.000	-0.411	-0.172
	4	-0.378	0.067	0.000	-0.522	-0.234
	5	-0.423	0.060	0.000	-0.553	-0.292
	6	-0.359	0.056	0.000	-0.480	-0.237
	7	-0.310	0.054	0.000	-0.427	-0.193
	8	-0.238	0.037	0.000	-0.319	-0.157
2	3	-0.149	0.036	0.001	-0.227	-0.072
	4	-0.236	0.046	0.000	-0.336	-0.136
	5	-0.281	0.042	0.000	-0.371	-0.191
	6	-0.217	0.041	0.000	-0.306	-0.128
	7	-0.168	0.035	0.000	-0.244	-0.093
	8	-0.097	0.030	0.006	-0.161	-0.032
3	4	-0.087	0.028	0.008	-0.146	-0.027
	5	-0.132	0.027	0.000	-0.190	-0.073
	6	-0.068	0.018	0.003	-0.107	-0.028
	7	-0.019	0.020	0.368	-0.062	0.025
	8	0.053	0.031	0.115	-0.015	0.120
4	5	-0.045	0.019	0.035	-0.086	-0.004
	6	0.019	0.027	0.482	-0.038	0.077
	7	0.068	0.026	0.021	0.012	0.124
	8	0.140	0.046	0.009	0.040	0.239
5	6	0.064	0.024	0.019	0.012	0.116
	7	0.113	0.024	0.000	0.061	0.165
	8	0.184	0.038	0.000	0.102	0.267
6	7	0.049	0.020	0.030	0.005	0.092
	8	0.120	0.028	0.001	0.061	0.180
7	8	0.072	0.031	0.038	0.005	0.138

Cortisol Day 2

(I) Phase	(J) Phase	Mean difference (I-J)	Std. Error	p level	95% CI	
					Lower limit	Higher limit
1	2	-0.082	0.028	0.011	-0.142	-0.022
	3	-0.235	0.042	0.000	-0.326	-0.145
	4	-0.294	0.047	0.000	-0.397	-0.192
	5	-0.376	0.049	0.000	-0.481	-0.271
	6	-0.269	0.047	0.000	-0.371	-0.168
	7	-0.262	0.030	0.000	-0.326	-0.198
	8	-0.135	0.055	0.029	-0.254	-0.016
2	3	-0.153	0.033	0.000	-0.224	-0.082
	4	-0.212	0.037	0.000	-0.293	-0.132
	5	-0.294	0.037	0.000	-0.373	-0.215
	6	-0.187	0.041	0.001	-0.276	-0.098
	7	-0.180	0.028	0.000	-0.240	-0.119
	8	-0.053	0.059	0.387	-0.180	0.075
3	4	-0.059	0.025	0.032	-0.112	-0.006
	5	-0.141	0.023	0.000	-0.191	-0.091
	6	-0.034	0.034	0.334	-0.107	0.039
	7	-0.027	0.030	0.397	-0.092	0.039
	8	0.100	0.058	0.106	-0.025	0.226
4	5	-0.082	0.017	0.000	-0.119	-0.044
	6	0.025	0.022	0.277	-0.023	0.074
	7	0.033	0.026	0.237	-0.024	0.089
	8	0.160	0.061	0.022	0.027	0.292
5	6	0.107	0.026	0.001	0.050	0.164
	7	0.114	0.029	0.002	0.051	0.177
	8	0.241	0.062	0.002	0.107	0.375
6	7	0.007	0.023	0.756	-0.042	0.056
	8	0.134	0.061	0.045	0.003	0.265
7	8	0.127	0.054	0.034	0.011	0.243

Chromogranin A Day 1

(I) Phase	(J) Phase	Mean difference (I-J)	Std. Error	p level	95% CI	
					Lower limit	Higher limit
1	2	-0.404	1.962	0.840	-4.642	3.835
	3	-0.997	1.875	0.604	-5.048	3.055
	4	-4.300	1.383	0.008	-7.289	-1.312
	5	-5.091	1.756	0.012	-8.886	-1.297
	6	-3.296	1.993	0.122	-7.601	1.010
	7	-1.417	1.542	0.375	-4.749	1.915
	8	-2.347	1.093	0.051	-4.708	0.014
2	3	-0.593	0.935	0.537	-2.613	1.428
	4	-3.897	1.828	0.053	-7.845	0.052
	5	-4.688	1.345	0.004	-7.594	-1.781
	6	-2.892	2.143	0.200	-7.522	1.739
	7	-1.014	2.085	0.635	-5.519	3.491
	8	-1.944	2.016	0.353	-6.298	2.411
3	4	-3.304	1.422	0.037	-6.376	-0.232
	5	-4.095	1.345	0.009	-7.000	-1.190
	6	-2.299	1.928	0.254	-6.464	1.865
	7	-0.421	1.820	0.821	-4.354	3.512
	8	-1.351	1.980	0.507	-5.629	2.928
4	5	-0.791	1.468	0.599	-3.961	2.379
	6	1.005	1.675	0.559	-2.615	4.624
	7	2.883	1.416	0.063	-0.176	5.942
	8	1.953	1.281	0.151	-0.815	4.721
5	6	1.796	1.635	0.292	-1.736	5.328
	7	3.674	1.325	0.016	0.812	6.535
	8	2.744	1.918	0.176	-1.400	6.888
6	7	1.878	1.140	0.123	-0.584	4.340
	8	0.948	1.945	0.634	-3.254	5.150
7	8	-0.930	1.762	0.607	-4.736	2.876

Chromogranin A Day 2

(I) Phase	(J) Phase	Mean difference (I-J)	Std. Error	p level	95% CI	
					Lower limit	Higher limit
1	2	-2.998	1.697	0.101	-6.664	0.668
	3	-4.166	1.578	0.020	-7.576	-0.756
	4	-4.660	1.767	0.021	-8.477	-0.842
	5	-5.597	1.549	0.003	-8.944	-2.250
	6	-5.389	1.627	0.006	-8.904	-1.875
	7	-1.747	0.937	0.085	-3.771	0.276
	8	-1.744	1.479	0.259	-4.938	1.451
2	3	-1.167	1.380	0.413	-4.150	1.815
	4	-1.662	1.702	0.347	-5.338	2.015
	5	-2.599	1.593	0.127	-6.040	0.842
	6	-2.391	1.492	0.133	-5.614	0.831
	7	1.251	1.755	0.489	-2.541	5.042
	8	1.254	2.098	0.560	-3.277	5.786
3	4	-0.494	0.955	0.614	-2.557	1.569
	5	-1.431	0.792	0.094	-3.142	0.279
	6	-1.224	1.198	0.326	-3.812	1.365
	7	2.418	1.380	0.103	-0.564	5.400
	8	2.422	2.401	0.332	-2.765	7.609
4	5	-0.937	0.498	0.082	-2.012	0.138
	6	-0.730	1.591	0.654	-4.167	2.708
	7	2.912	1.595	0.091	-0.533	6.358
	8	2.916	2.705	0.301	-2.927	8.759
5	6	0.208	1.334	0.879	-2.674	3.090
	7	3.850	1.491	0.023	0.628	7.072
	8	3.853	2.397	0.132	-1.325	9.031
6	7	3.642	1.613	0.042	0.158	7.126
	8	3.646	2.060	0.100	-0.806	8.097
7	8	0.004	1.985	0.999	-4.285	4.293

Memory Day 1

(I) Loop	(J) Loop	Mean difference (I-J)	Std. Error	p level	95% CI	
					Lower limit	Higher limit
1	2	-1.071	0.425	0.026	-1.990	-0.153
	3	0.857	0.653	0.212	-0.555	2.269
	4	0.000	0.734	1.000	-1.585	1.585
2	3	1.929	0.633	0.009	0.561	3.296
	4	1.071	0.699	0.149	-0.439	2.582
3	4	-0.857	0.467	0.089	-1.866	0.152

Memory Day 2

(I) Loop	(J) Loop	Mean difference (I-J)	Std. Error	p level	95% CI	
					Lower limit	Higher limit
1	2	0.500	0.454	0.291	-0.481	1.481
	3	2.286	0.549	0.001	1.100	3.472
	4	1.429	0.571	0.027	0.194	2.663
2	3	1.786	0.595	0.010	0.501	3.070
	4	0.929	0.518	0.097	-0.192	2.049
3	4	-0.857	0.553	0.145	-2.052	0.338

Visual attention Day 1

(I) Phase	(J) Phase	Mean difference (I-J)	Std. Error	p level	95% CI	
					Lower limit	Higher limit
1	2	-2.643	1.239	0.052	-5.319	0.033
	3	-4.357	1.269	0.004	-7.099	-1.615
	4	-6.071	1.396	0.001	-9.088	-3.055
	5	-4.571	1.073	0.001	-6.889	-2.254
	6	-7.571	1.540	0.000	-10.898	-4.245
2	3	-1.714	1.056	0.128	-3.995	0.567
	4	-3.429	1.026	0.005	-5.644	-1.213
	5	-1.929	1.112	0.106	-4.330	0.473
	6	-4.929	1.220	0.001	-7.564	-2.293
3	4	-1.714	0.986	0.106	-3.844	0.415
	5	-0.214	0.459	0.648	-1.206	0.777
	6	-3.214	1.223	0.021	-5.857	-0.572
4	5	1.500	0.924	0.129	-0.497	3.497
	6	-1.500	1.088	0.191	-3.851	0.851
5	6	-3.000	1.186	0.025	-5.562	-0.438

Visual attention Day 2

(I) Phase	(J) Phase	Mean difference (I-J)	Std. Error	p level	95% CI	
					Lower limit	Higher limit
1	2	-2.214	1.100	0.065	-4.591	0.163
	3	-0.929	0.848	0.293	-2.761	0.904
	4	-2.929	0.946	0.009	-4.973	-0.885
	5	-0.929	0.730	0.226	-2.505	0.648
	6	-2.143	0.882	0.030	-4.049	-0.237
2	3	1.286	1.019	0.229	-0.915	3.486
	4	-0.714	0.854	0.418	-2.560	1.132
	5	1.286	0.922	0.187	-0.707	3.278
	6	0.071	0.722	0.923	-1.489	1.632
3	4	-2.000	1.084	0.088	-4.343	0.343
	5	0.000	0.914	1.000	-1.974	1.974
	6	-1.214	0.921	0.210	-3.204	0.775
4	5	2.000	1.144	0.104	-0.470	4.470
	6	0.786	0.757	0.318	-0.850	2.422
5	6	-1.214	0.786	0.146	-2.912	0.483

Attention/mental flexibility Day 1

(I) Phase	(J) Phase	Mean difference (I-J)	Std. Error	p level	95% CI	
					Lower limit	Higher limit
1	2	3.143	1.955	0.132	-1.081	7.367
	3	8.786	1.989	0.001	4.488	13.084
	4	11.000	2.226	0.000	6.191	15.809
	5	11.571	2.301	0.000	6.601	16.542
	6	9.500	2.629	0.003	3.821	15.179
2	3	5.643	1.369	0.001	2.685	8.601
	4	7.857	2.463	0.007	2.537	13.177
	5	8.429	2.005	0.001	4.098	12.759
	6	6.357	2.286	0.016	1.418	11.296
3	4	2.214	1.849	0.253	-1.781	6.209
	5	2.786	2.209	0.230	-1.987	7.559
	6	0.714	2.590	0.787	-4.881	6.310
4	5	0.571	3.063	0.855	-6.046	7.188
	6	-1.500	3.038	0.630	-8.063	5.063
5	6	-2.071	3.211	0.530	-9.009	4.866

Attention/mental flexibility Day 2

(I) Phase	(J) Phase	Mean difference (I-J)	Std. Error	p level	95% CI	
					Lower limit	Higher limit
1	2	9.500	2.942	0.007	3.144	15.856
	3	8.214	3.173	0.022	1.359	15.069
	4	9.786	2.798	0.004	3.742	15.829
	5	12.143	2.518	0.000	6.704	17.582
	6	11.143	3.305	0.005	4.003	18.282
2	3	-1.286	2.579	0.626	-6.858	4.287
	4	0.286	1.920	0.884	-3.862	4.433
	5	2.643	1.750	0.155	-1.137	6.423
	6	1.643	1.929	0.410	-2.524	5.810
3	4	1.571	2.639	0.562	-4.130	7.272
	5	3.929	1.659	0.034	0.344	7.513
	6	2.929	3.239	0.382	-4.068	9.925
4	5	2.357	1.737	0.198	-1.396	6.110
	6	1.357	2.548	0.603	-4.147	6.861
5	6	-1.000	3.024	0.746	-7.532	5.532

Functional psychobiosocial states Day 1

(I) Phase	(J) Phase	Mean difference (I-J)	Std. Error	p level	95% CI	
					Lower limit	Higher limit
1	2	-0.071	0.099	0.484	-0.285	0.143
	3	0.082	0.088	0.372	-0.109	0.272
	4	-0.061	0.227	0.792	-0.553	0.430
	5	-0.194	0.202	0.354	-0.630	0.242
	6	-0.194	0.181	0.304	-0.586	0.198
	7	-0.347	0.125	0.016	-0.617	-0.077
	8	-0.408	0.175	0.037	-0.787	-0.030
2	3	0.153	0.092	0.119	-0.045	0.351
	4	0.010	0.206	0.961	-0.434	0.454
	5	-0.122	0.209	0.567	-0.573	0.328
	6	-0.122	0.171	0.486	-0.491	0.246
	7	-0.276	0.157	0.102	-0.614	0.063
	8	-0.337	0.162	0.058	-0.686	0.013
3	4	-0.143	0.158	0.384	-0.485	0.200
	5	-0.276	0.142	0.074	-0.582	0.030
	6	-0.276	0.142	0.074	-0.582	0.030
	7	-0.429	0.126	0.005	-0.701	-0.156
	8	-0.490	0.180	0.018	-0.879	-0.101
4	5	-0.133	0.121	0.293	-0.394	0.129
	6	-0.133	0.138	0.353	-0.430	0.165
	7	-0.286	0.181	0.138	-0.677	0.105
	8	-0.347	0.236	0.166	-0.857	0.163
5	6	0.000	0.186	1.000	-0.403	0.403
	7	-0.153	0.168	0.380	-0.517	0.211
	8	-0.214	0.234	0.377	-0.720	0.292
6	7	-0.153	0.119	0.222	-0.411	0.105
	8	-0.214	0.169	0.226	-0.579	0.150
7	8	-0.061	0.133	0.653	-0.348	0.226

Functional psychobiosocial states Day 2

(I) Phase	(J) Phase	Mean difference (I-J)	Std. Error	p level	95% CI	
					Lower limit	Higher limit
1	2	-0.029	0.090	0.755	-0.222	0.165
	3	-0.049	0.115	0.677	-0.297	0.199
	4	0.063	0.159	0.697	-0.279	0.406
	5	-0.080	0.177	0.660	-0.462	0.302
	6	-0.161	0.142	0.277	-0.468	0.146
	7	-0.049	0.107	0.654	-0.280	0.182
	8	-0.049	0.146	0.742	-0.364	0.266
2	3	-0.020	0.080	0.804	-0.194	0.153
	4	0.092	0.122	0.466	-0.172	0.356
	5	-0.051	0.156	0.749	-0.388	0.286
	6	-0.133	0.149	0.391	-0.455	0.190
	7	-0.020	0.105	0.848	-0.247	0.206
	8	-0.020	0.156	0.898	-0.356	0.316
3	4	0.112	0.071	0.136	-0.040	0.265
	5	-0.031	0.114	0.793	-0.277	0.216
	6	-0.112	0.134	0.418	-0.402	0.177
	7	0.000	0.090	1.000	-0.194	0.194
	8	0.000	0.151	1.000	-0.327	0.327
4	5	-0.143	0.109	0.213	-0.378	0.093
	6	-0.224	0.146	0.147	-0.539	0.090
	7	-0.112	0.096	0.264	-0.320	0.095
	8	-0.112	0.166	0.511	-0.471	0.247
5	6	-0.082	0.107	0.458	-0.312	0.149
	7	0.031	0.121	0.804	-0.231	0.292
	8	0.031	0.166	0.857	-0.328	0.390
6	7	0.112	0.094	0.253	-0.090	0.315
	8	0.112	0.148	0.461	-0.207	0.431
7	8	0.000	0.102	1.000	-0.219	0.219

Dysfunctional psychobiosocial states Day 1

(I) Phase	(J) Phase	Mean difference (I-J)	Std. Error	p level	95% CI	
					Lower limit	Higher limit
1	2	-0.104	0.054	0.076	-0.221	0.013
	3	-0.160	0.064	0.026	-0.298	-0.023
	4	-0.244	0.068	0.003	-0.391	-0.096
	5	-0.205	0.071	0.013	-0.358	-0.052
	6	-0.192	0.117	0.125	-0.445	0.061
	7	-0.163	0.084	0.075	-0.344	0.019
	8	0.047	0.156	0.769	-0.291	0.385
2	3	-0.056	0.043	0.218	-0.150	0.038
	4	-0.140	0.050	0.016	-0.248	-0.031
	5	-0.101	0.069	0.168	-0.250	0.048
	6	-0.088	0.110	0.439	-0.326	0.150
	7	-0.059	0.100	0.568	-0.275	0.158
	8	0.151	0.166	0.380	-0.208	0.510
3	4	-0.083	0.059	0.181	-0.211	0.044
	5	-0.044	0.074	0.557	-0.204	0.115
	6	-0.032	0.107	0.772	-0.263	0.200
	7	-0.002	0.095	0.981	-0.207	0.202
	8	0.207	0.181	0.273	-0.183	0.598
4	5	0.039	0.064	0.552	-0.099	0.176
	6	0.052	0.120	0.673	-0.207	0.310
	7	0.081	0.099	0.429	-0.133	0.295
	8	0.291	0.174	0.119	-0.086	0.667
5	6	0.013	0.095	0.895	-0.192	0.218
	7	0.042	0.094	0.660	-0.160	0.244
	8	0.252	0.147	0.111	-0.067	0.570
6	7	0.029	0.118	0.807	-0.225	0.284
	8	0.239	0.168	0.178	-0.123	0.601
7	8	0.210	0.162	0.219	-0.141	0.560

Dysfunctional psychobiosocial states Day 2

(I) Phase	(J) Phase	Mean difference (I-J)	Std. Error	p level	95% CI	
					Lower limit	Higher limit
1	2	-0.075	0.033	0.041	-0.147	-0.003
	3	-0.109	0.060	0.090	-0.238	0.019
	4	-0.269	0.082	0.006	-0.447	-0.091
	5	-0.163	0.071	0.040	-0.317	-0.009
	6	-0.100	0.100	0.334	-0.315	0.115
	7	-0.200	0.083	0.031	-0.379	-0.022
	8	-0.064	0.094	0.506	-0.267	0.138
2	3	-0.034	0.056	0.551	-0.155	0.086
	4	-0.194	0.086	0.042	-0.379	-0.008
	5	-0.088	0.064	0.193	-0.226	0.050
	6	-0.025	0.089	0.786	-0.216	0.167
	7	-0.125	0.093	0.204	-0.327	0.077
	8	0.011	0.088	0.901	-0.179	0.201
3	4	-0.160	0.068	0.035	-0.306	-0.013
	5	-0.054	0.064	0.418	-0.192	0.085
	6	0.010	0.080	0.907	-0.164	0.183
	7	-0.091	0.081	0.283	-0.266	0.084
	8	0.045	0.089	0.620	-0.147	0.237
4	5	0.106	0.088	0.250	-0.084	0.296
	6	0.169	0.104	0.128	-0.056	0.394
	7	0.069	0.081	0.410	-0.105	0.243
	8	0.205	0.082	0.027	0.027	0.382
5	6	0.063	0.055	0.272	-0.056	0.182
	7	-0.037	0.090	0.686	-0.232	0.158
	8	0.099	0.100	0.342	-0.118	0.315
6	7	-0.101	0.088	0.277	-0.292	0.091
	8	0.036	0.094	0.710	-0.167	0.238
7	8	0.136	0.077	0.102	-0.031	0.303
